# Loneliness, Sleep Quality, and Cognitive Function in Community-Dwelling Older Adults

**DOI:** 10.1093/geroni/igab046.655

**Published:** 2021-12-17

**Authors:** Kexin Yu, Bernadette Fausto

**Affiliations:** 1 USC, LA, California, United States; 2 Rutgers University–Newark, Newark, New Jersey, United States

## Abstract

Loneliness is a risk factor for cognitive decline in older adults, however, the underlying mechanisms are less understood. Individuals who experience frequent loneliness tend to have poorer sleep quality. Empirical evidence supports the influence of sleep on cognitive health. This study examined the possible mediating effect of sleep characteristics on the relationship between loneliness and cognition. The study sample included 557 participants from wave 2 of the National Social Life, Health, and Aging Project who had actigraphy sleep measures (mean age = 73.17, 52.6% female). Loneliness was assessed with the 3-item UCLA Loneliness Scale. Cognitive function was measured with the Montreal Cognitive Assessment. Five sleep quality indicators were objectively recorded with wearable devices: assumed sleep time; actigraphy sleep time; time spent awake after sleep onset (WASO); sleep fragmentation; and sleep percentage (actigraphy sleep/(assumed sleep + WASO)). Path analysis model results show that WASO, fragmentation, and sleep percentage mediate the link between loneliness and cognitive function. Loneliness was positively related to WASO, and WASO was negatively associated with cognition. Loneliness correlated with increased sleep fragmentation which was associated with worse cognitive function. Individuals who had more frequent loneliness had a lower sleep percentage, and sleep percentage was positively associated with cognitive function. Nonetheless, the path from loneliness to these three sleep characteristics became insignificant after controlling for depressive symposiums. Depressive symptoms and fragmentation were found to double mediate the association between loneliness and cognitive function. Sleep and depression could be underlying pathways for the association between loneliness and cognition.

